# The Limits of Linked Suppression for Regulatory T Cells

**DOI:** 10.3389/fimmu.2016.00082

**Published:** 2016-03-09

**Authors:** Toshiro Ito, Akira Yamada, Ibrahim Batal, Melissa Y. Yeung, Martina M. McGrath, Mohamed H. Sayegh, Anil Chandraker, Takuya Ueno

**Affiliations:** ^1^Transplantation Unit, Surgical Services, Massachusetts General Hospital, Harvard Medical School, Boston, MA, USA; ^2^Transplantation Research Center, Brigham and Women’s Hospital and Children’s Hospital, Harvard Medical School, Boston, MA, USA

**Keywords:** costimulation, indirect pathway, MHC class II, tolerance, regulatory T cells

## Abstract

**Background:**

We have previously found that CD4^+^CD25^+^ regulatory T cells (Tregs) can adoptively transfer tolerance after its induction with costimulatory blockade in a mouse model of murine cardiac allograft transplantation. In these experiments, we tested an hypothesis with three components: (1) the Tregs that transfer tolerance have the capacity for linked suppression, (2) the determinants that stimulate the Tregs are expressed by the indirect pathway, and (3) the donor peptides contributing to these indirect determinants are derived from donor major histocompatibility complex (MHC) antigens (Ags).

**Methods:**

First heart transplants were performed from the indicated donor strain to B10.D2 recipients along with costimulatory blockade treatment (250 μg i.p. injection of MR1 on day 0 and 250 μg i.p. injection of CTLA-4 Ig on day 2). At least 8 weeks later, a second heart transplant was performed to a new B10.D2 recipient who had been irradiated with 450 cGy. This recipient was given 40 × 106 naive B10.D2 spleen cells + 40 × 106 B10.D2 spleen cells from the first (tolerant) recipient. We performed three different types of heart transplants using various donors.

**Results:**

(1) Tregs suppress the graft rejection in an Ag-specific manner. (2) Tregs generated in the face of MHC disparities suppress the rejection of grafts expressing third party MHC along with tolerant MHC.

**Conclusion:**

The limits of linkage appear to be quantitative and not universally determined by either the indirect pathway or by peptides of donor MHC Ags.

## Introduction

The physiologically unusual stimulation of T cells by donor antigen-presenting cells (APCs) has been called “direct” recognition, whereas stimulation by self-APCs, presenting peptides of donor origin, has been called “indirect” recognition. Direct recognition has been believed to be the major pathway involved in allograft rejection due to three basic observations, namely, (1) direct stimulation is very strong in a primary allogenic mixed lymphocyte reaction, (2) depletion of donor APCs can sometimes prolong allograft survival, and (3) donor major histocompatibility complex (MHC) antigens (Ags) are more important than minor Ags in causing graft rejection (1). Matching for MHC Ags achieves better allograft survival. Lechler and Batchelor showed the importance of MHC class II matching compared to MHC class I matching at least in the long-term survival (2). However, there are several remarkable reports of consequences of T cells responding via the indirect pathway. These reports showed the indirect pathway (a) helps for priming alloreactive CD8 T cells (3, 4), (b) is essential for tolerance induction in some models (5, 6), and (c) is involved in chronic transplant rejection (7, 8). In addition, several papers have shown the importance of an indirect response in allograft rejection (1, 3, 9, 10). Indirect allorecognition contributes not only to acute graft rejection (2, 9) but also possibly to the continuing response to the allograft in the long term after transplantation (11). Previously, we tested the role of costimulatory blockade for prolonging allograft survival with using class II-deficient mice when only one or the other pathway of graft rejection was available. We found that to achieve long-term survival after costimulatory blockade requires that the recipient expresses MHC class II molecules (12). This result indicated that indefinite cardiac transplant survival could not be achieved in the absence of an intact indirect pathway. These results are consistent with the fact that at least a component of the regulatory T cell (Treg) response must involve recognition of peptides of donor Ags presented by recipient MHC molecules (13). Authors also mentioned that linked suppression can also be induced through the indirect pathway. However, little work seems to have addressed their direct role in transplantation. Therefore, in the current study, we tested whether the Tregs that transfer tolerance have the capacity for linked suppression.

## Methods and Results

**Figure 1:**
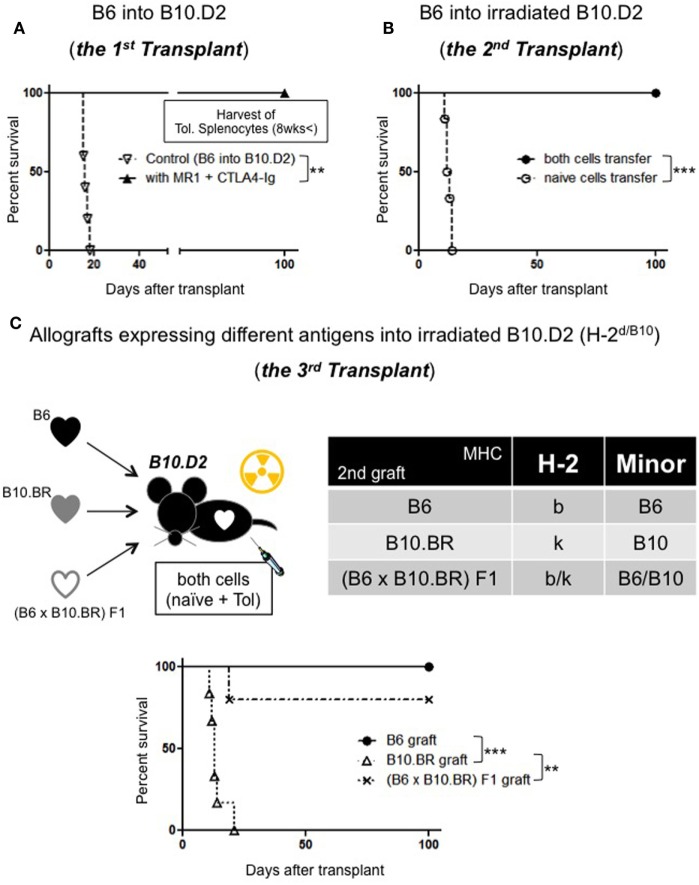
**(A) Allograft survival in B10.D2 recipients**: (>100 days, *n* = 5, *p* = 0.0017 compared to control: 16.2 days, *n* = 5). **(B)** **Adoptive transfer model**: naive splenocytes transfer (*n* = 6), both naive and Tol. splenocytes transfer (100 days, *n* = 6, *p* = 0.0007). **(C)** **Linkage model**: B6 hearts (>100 days), B10.BR hearts (~23 days, *n* = 6, *p* = 0.0006) (B6 × B10.BR) F1 (>100 days, *n* = 4/5, *p* = 0.0044).

First, we made B10.D2 (H-2^d^) mice tolerant to B6 (H-2^b^) with costimulatory blockade [250 μg intraperitoneal (i.p.) injection of MR1 on day 0 and 250 μg i.p. injection of CTLA-4 Ig on day 2] (Figure 1A). At least 8 weeks later, a second heart transplant was performed to a new B10.D2 recipient who had been irradiated with 450 cGy. All recipient received intravenous (i.v.) injection of naive 40 × 10^6^ splenocytes + 40 × 10^6^ splenocytes that are taken from the tolerant mice (toleralized splenocytes: Tol.) significantly prolonged graft survival compared to recipient received only naive splenocyte (12 ± 1 days compared to >100, *p* < 0.001) (Figure 1B). After these results, we considered linkage of Tregs. Next, we performed a second transplant from B6 mice to irradiated B10.D2 mice. The second donors express the same MHC and minor Ags as the first graft or B10.BR heart grafts differ from the first graft in their MHC Ags or (B6 × B10.BR) F1 mice, which express both H-2^b^ and H-2^k^ Ags. After transplant, the mice received i.v. injection of naive and toleralized splenocytes. All B6 hearts survived over 100 days. But B10.BR hearts expressing third party MHC were rejected by 23 days (Figure 1C). (B10.BR × B6) F1 hearts expressing third party MHC with tolerant MHC showed 80% survival of over 100 days; however, CAV was observed in some specimen. The institutional subcommittee on research animal care at Massachusetts General Hospital approved all animal experiments.

## Discussion

Linked suppression has often been associated with Tregs, and its mechanisms must be important ones, as tolerance can be extended to whole MHC disparities when applied to cardiac transplantation. Tolerance was extended to third party transplant Ags, even to MHC-encoded Ags, provided they are expressed on the same graft as the tolerated Ags in some models (14–17). Thus, its mechanism of immunoregulation in transplantation is very important. In addition, understanding interactions between linked suppression and Tregs can potentially be great advantage in the setting of transplantation to propagate the development of specific unresponsiveness once the process has been initiated.

Our preliminary data showed that Tregs suppress the graft rejection in an Ag-specific manner and Tregs generated in the face of MHC disparities suppress the rejection of grafts expressing third party MHC along with tolerant MHC.

## Conclusion

The very limited comparison in this experiment will determine whether the patterns of gene expression can reliably distinguish a regulatory population from one that promotes rejection.

## Author Contributions

TU, TI, and AY participated in the performance of the research, performed the data collection, performed the statistical analysis, and contributed to the writing of the manuscript; IB, MY, and MM participated in the writing of the manuscript and performed review; and MS and TU designed the study and participated in review. AC participated in review.

## Conflict of Interest Statement

The authors declare that the research was conducted in the absence of any commercial or financial relationships that could be construed as a potential conflict of interest.

## Abbreviations

Ags, antigens; APCs, antigen-presenting cells; MHC, major histocompatibility complex; MST, median survival time; Tregs, regulatory T cells.
